# Effects of glycyrrhizin on healing and prevention of recurrent aphthous stomatitis in hamster models

**DOI:** 10.1371/journal.pone.0338806

**Published:** 2026-01-16

**Authors:** Fumie Shiba, Shiiko Maekawara, Hisako Furusho, Eri Ishida, Hideo Shigeishi, Kouji Ohta, Mutsumi Miyauchi

**Affiliations:** 1 Collaborative Research Laboratory of Oral Inflammation Regulation, Graduate School of Biomedical and Health Sciences, Hiroshima University, Hiroshima, Japan; 2 Faculty of Dentistry, Hiroshima University, Hiroshima, Japan; 3 Department of Oral and Maxillofacial Pathobiology, Graduate School of Biomedical and Health Sciences, Hiroshima University, Hiroshima, Japan; 4 Department of Advanced Prosthodontics, Graduate School of Biomedical and Health Sciences, Hiroshima University, Hiroshima, Japan; 5 Department of Public Oral Health, Program of Oral Health Sciences, Graduate School of Biomedical and Health Sciences, Hiroshima University, Hiroshima, Japan; Sungkyunkwan University - Suwon Campus: Sungkyunkwan University - Natural Sciences Campus, KOREA, REPUBLIC OF

## Abstract

Recurrent aphthous stomatitis (RAS), a major type of stomatitis, can significantly impair quality of life. The therapeutic and preventive effects of glycyrrhizin (GL), a compound known for its anti-inflammatory properties, remain unclear due to the lack of appropriate animal models, especially for prevention studies. Therefore, this study aimed to evaluate the therapeutic and preventive effects of GL and determine the optimal concentrations using two hamster models (stomatitis-initiation model and stomatitis-healing model) representing the initiation and healing phases of RAS. The effects were evaluated through macroscopic and histological analyses, gene expression profiling in hamster buccal tissues, and prostaglandin E_2_ (PGE_2_) assays in lipopolysaccharide-stimulated human oral keratinocytes. In the stomatitis-healing model, a low concentration of GL (0.0065%) significantly increased the cure rate and histologically reduced the numbers of vessels and lymphocytes. In the stomatitis-initiation model, low concentrations of GL (0.0065% and 0.033%) significantly decreased the edema score and histologically reduced the numbers of vessels and neutrophils, as well as the mRNA expression levels of *interleukin-6* and *cyclooxygenase-2*. In contrast, a high concentration of GL (0.33%) showed inferior efficacy compared with low concentrations in both models. Similarly, in LPS-stimulated human oral keratinocytes, low GL concentrations suppressed PGE₂ protein expression, while the highest concentration increased it. These findings show that GL promotes healing and prevents the onset of stomatitis at specific concentrations, underscoring the importance of optimal dosing and supporting the potential clinical application of GL in the management of RAS.

## Introduction

The oral cavity serves as the entry point to the digestive system and plays a critical role in sustaining life through food intake and chewing. Oral diseases can significantly affect overall health and well-being and have emerged as a major global health concern. Stomatitis, a common oral condition, can severely impair quality of life. Typical symptoms include pain, bleeding, ulceration, difficulty swallowing liquids and solids, and difficulty speaking [1–3].

Stomatitis refers to inflammation of the oral mucosa, which can arise from various causes such as mechanical irritation from dentures and orthodontic appliances, radiation therapy, chemotherapy, bacterial and viral infections, stress, heredity, and fatigue [4,5]. Depending on the clinical presentation, stomatitis can be classified into different types. One major type is recurrent aphthous stomatitis (RAS), which affects approximately 20% of the population [6].

RAS is characterized by recurrent, painful, solitary, or multiple small ulcers with erythematous halos and yellow/gray floors within the oral cavity [7]. The clinical management of RAS primarily involves topical therapies, including corticosteroids [8], topical antibiotics [9,10], and topical anesthetics [11]. However, these treatments can be associated with serious side effects. Use of a toothpaste that does not contain sodium lauryl sulfate (SLS) [12] and supplementation with vitamin B_12_ are considered effective methods of preventing RAS [13,14]; however, no definitive prevention of RAS has been established.

The pathophysiology underlying the development of RAS is poorly understood, although inflammatory cell infiltration is widely considered to substantially contribute to its onset and exacerbation [4]. Consequently, agents that inhibit inflammatory cell infiltration may offer potential therapeutic benefits in preventing or mitigating the development of stomatitis.

Glycyrrhizin (GL), the principal bioactive compound isolated from licorice [15], exerts potent anti-inflammatory effects primarily by inhibiting the high mobility group box 1 (HMGB1) – toll-like receptor 4 (TLR4) – nuclear factor-kappa B (NF-κB) signaling pathway and modulating arachidonic acid metabolism, thereby suppressing pro-inflammatory cytokines such as interleukin-6 (IL-6) and tumor necrosis factor alpha (Tnf-α) [16–18]. In the oral field, GL is incorporated into oral formulations and is widely used to suppress inflammation such as gingivitis and periodontitis [19]. Beyond the oral cavity, animal studies have demonstrated GL’s skin wound healing [20]. Additionally, GL has shown not only therapeutic but also preventive effects in ulcerative colitis (UC) models, where it attenuates disease development by suppressing inflammatory cell infiltration and mucosal damage [21–23]. Although UC and RAS occur in different anatomical sites, they share common pathological features, including epithelial barrier disruption, bursts of inflammatory cytokines, and abnormal activation of inflammatory cells. Based on these similarities, we hypothesized that GL may exert not only therapeutic but also preventive effects against RAS, as observed in UC.

In the present study, we developed a stomatitis-healing model to assess the healing process and a novel stomatitis-initiation model to evaluate the early phases of stomatitis development (from the initiation phase to signal amplification [24,25], in which inflammatory cell infiltration, edema, vasodilation, and epithelial degeneration/disorder occur). Most existing stomatitis models reproduce the ulcerative stage, which corresponds to a phase beyond the early stage of stomatitis onset that is crucial for prevention studies. Therefore, in this study, we newly established the stomatitis-initiation model that can trace the early process of stomatitis development, enabling the evaluation of drug effects on stomatitis prevention.

This study aimed to examine the potential of GL in promoting stomatitis healing and onset prevention using the stomatitis-healing and initiation models.

## Materials and methods

### Reagents

We used potassium salt of GL (Dipotassium glycyrrhizinate; Alps Pharmaceutical, Inc. Co., Ltd. Gifu, Japan) that ionizes glycyrrhizin in water. Acetic acid was purchased from Sigma-Aldrich (St. Louis, MO, USA). The acetic acid and GL solutions used in the experiments were prepared in physiological saline (PS) (OTSUKA NORMAL SALINE, Otsuka Pharmaceutical Factory, Inc. Tokyo, Japan). The concentrations of GL were set at three levels within the range relevant to oral stomatitis therapeutics and oral care products. For each experiment, PS was administered as the vehicle control (ST/PS and STI/PS). LPS from *Escherichia coli (E.coli*-LPS: B6) was purchased from Sigma-Aldrich (St. Louis, MO, USA).

### Ethics statement

All experiments described herein were approved by Hiroshima University’s Ethics Committee for Animal Experimentation (Permit Number: A20-45) and were performed in accordance with the “Guidelines for the Care and Use of Laboratory Animals” established by Hiroshima University, in accordance with the ARRIVE [26] and AVMA guidelines. The sample size was constrained by ethical considerations and financial feasibility, but was sufficient to observe consistent trends across experimental groups.

### Animals

Overall, 104 5-week-old male golden Syrian hamsters (*Mesocricetus auratus*) weighing 92.1 ± 6.1 g (76.4–104.2 g) (S1 Table) (Charles River Japan, Inc., Yokohama, Japan) were housed in a specific pathogen-free facility in 12 h light-dark cycles with ad libitum access to water and food and kept at a constant ambient temperature and humidity (22 °C, 50 ± 5% RH). The maximum number of animals per cage was three. To prevent grooming contact with other groups, each group was kept in its own cage.

During the experiment, the hamsters were anesthetized via intraperitoneal injection of a mixture of three anesthetics (3 mL/kg/animal)—Medetomidine hydrochloride (0.03 mg/mL; Dorbene® vet, Kyoritsuseiyaku Co., Tokyo, Japan), Midazolam (0.4 mg/mL; Midazolam Injection 10 mg, Sandoz K.K., Tokyo, Japan), and Butorphanol tartrate (0.5 mg/mL; Vetorphale®, Meiji Seika Pharma Co., Ltd., Tokyo, Japan) adjusted with saline (OTSUKA NORMAL SALINE, Otsuka Pharmaceutical Factory, Inc. Tokyo, Japan). All efforts were made to minimize animal suffering. The hamsters were placed and fixed on their backs on an experimental stand. After the experiment, all animals were euthanized using CO_2_ gas.

### Stomatitis-healing model

**Fig 1:**
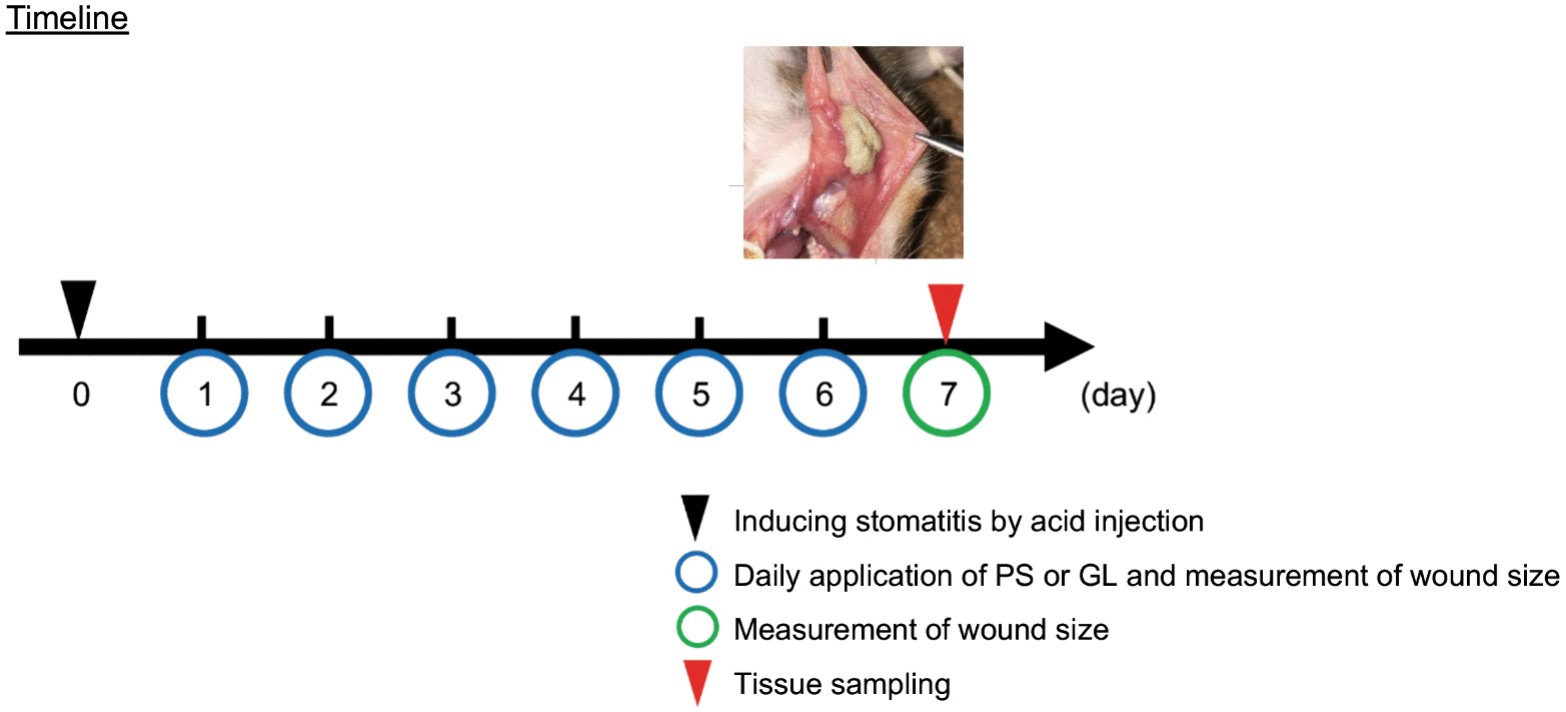
The timeline of the stomatitis-healing model. Schematic representation of the experimental schedule from day 0 to day 7. PS: physiological saline; GL: glycyrrhizin.

The timeline of the experiment is shown in Fig 1.

The established stomatitis-healing model was used for evaluation [27]. Under anesthesia, the right cheek pouches of the hamsters were extended outside the oral cavity. 30 µL of 10% acetic acid solution was injected into the submucosal connective tissue of the cheek pouch using a microsyringe (BD Lo-Dose ^TM^ U-100 Insulin Syringe, 1/2 mL, 30 G, 8 mm, Nippon Becton Dickinson Co., Ltd. Tokyo, Japan). The cheek pouch was replaced in the oral cavity. Starting from the day after stomatitis induction, 50 µL of different concentrations of GL (0.0065%, 0.033%, and 0.33%) were applied once daily for seven consecutive days, while PS (50 µL) was applied to the lesions in the control group. Using a microcaliper, the long and short diameters of the wound were measured daily for 7 days, and the area of the wound was determined by multiplying the two values. The healing efficacy of each wound was evaluated by comparing the cure rates over time. The cure rate was calculated using the following formula [27]:

$\text{Cure}\;\text{rate}\;(\%)=\frac{\text{wound}\;\text{area}\;\text{of}\;\text{d1}\;\text{mesaurement}\;–\;\text{wound}\;\text{area}\;\text{of}\;\text{dn}\;\text{measurement}}{\text{wound}\;\text{area}\;\text{of}\;\text{d1}\;\text{measurement}}\times \text{1}\text{0}\text{0}$

Here, d1 represents the wound area on the day after acetic acid injection, and dn represents the wound area on day n after acetic acid injection. Exactly 7 days after acetic acid injection, hamsters from each group were euthanized, their cheek pouches were removed, and tissue sections were prepared for histological evaluation.

### Measurement of the hamster’s weight

Previous studies have indicated a possible association between body weight gain and stomatitis-related pain in growing hamsters [27]. The following formula was used to determine the amount of weight gain from the day after acetic acid injection [27]: Percent weight gain (%) = [(dn - d1)/d1] × 100, where d1 is the hamster’s weight on the day after acetic acid injection, and dn is the hamster’s weight recorded n days after acetic acid injection.

### Establishment of the novel stomatitis-initiation model

**Fig 2:**
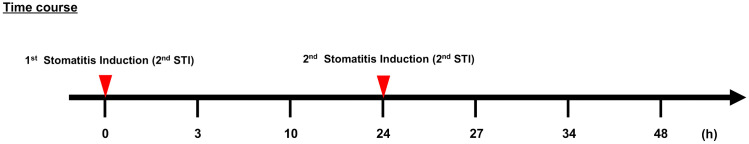
Time course after stomatitis induction. Healthy control (0 h, without stomatitis induction (STI)) and time points after stomatitis induction: 3 h after the first STI, 10 h after the first STI, 24 h after the first STI (just before the second STI), 27 h (3 h after the second STI), 34 h (10 h after the second STI), 48 h (24 h after the second STI).

The timeline of the experiment is shown in Fig 2. There were seven observation and tissue collection time points (n = 5). A total of 35 5-week-old hamsters were used in this study. Five hamsters were in the healthy group that received a single administration of PS without stomatitis induction. The remaining 30 hamsters were fixed on a cork board under anesthesia, and a 6-mm diameter filter paper (Advantec TOYO, 5B, Tokyo, Japan) immersed in 30% acetic acid solution was attached to the right buccal mucosa for 10 s to induce stomatitis (inflammatory edema beneath the covering mucosal epithelium). PS was administered just before stomatitis induction. Five hamsters each were euthanized 3, 10, and 24 h after stomatitis induction, and the buccal mucosal tissues were collected, divided into two parts, and used for genetic analysis and histological evaluation after macroscopic observation.

After 24 h, the same operation was repeated to induce stomatitis. PS was administered just before stomatitis induction. Five hamsters each were euthanized 3, 10, and 24 h after stomatitis induction, and the buccal mucosal tissues were collected, divided into two parts, and used for genetic analysis and histological evaluation after macroscopic observation.

### Evaluation of the effects of GL using the stomatitis-initiation model

Stomatitis is known to progress through stepwise morphological changes in the epithelium and submucosal tissue, beginning with erythema and surface irregularity (clinically recognized as “roughness”), followed by edema and inflammatory cell infiltration, and ultimately leading to ulcer formation [28,29].

We developed a novel stomatitis-initiation model to evaluate the early phases of stomatitis development, which are characterized by mucosal surface roughening followed by edema. These phases range from the initiation stage to signal amplification [24,25], during which inflammatory cell infiltration, edema, vasodilation, and epithelial degeneration/disorder occur.

**Fig 3:**
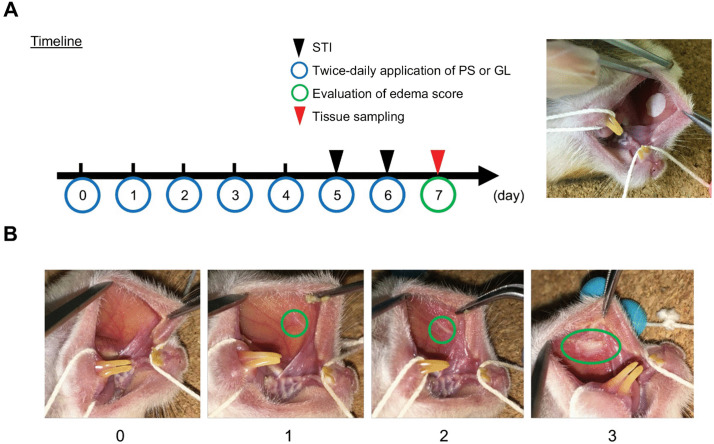
Timeline of the stomatitis-initiation model and the edema score. **(A)** Timeline of the stomatitis-initiation model. Schematic representation of the experimental schedule from day 0 to day 7. PS: physiological saline; GL: glycyrrhizin. **(B)** The degree of edema was visually evaluated on a 4-point scale (0–3) according to the edema score: **0:** Healthy buccal mucosa with a glossy surface, without roughness/atrophy, erythema, or inflammatory edema. **1:** Mucosal surface showing roughness/atrophy with accompanying erythema, but no inflammatory edema. **2:** Presence of inflammatory edema, with an area smaller than that of acetic acid–impregnated filter paper (diameter: 6 mm). **3:** Presence of inflammatory edema, with an area larger than that of acetic acid–impregnated filter paper.

Using the novel stomatitis-initiation model, the effect of GL solution was evaluated based on the timeline shown in Fig 3A.

50 µL of different GL concentrations (0.0065%, 0.033%, and 0.33%) were topically applied to the right buccal mucosa of hamsters twice a day for 6 consecutive days. The first and second applications were separated by at least 3 h. On days 5 and 6, in parallel with the following operation, hamsters were fixed under anesthesia, and stomatitis was induced. GL solutions were administered just before and after stomatitis induction. On day 6 and day 7, the degree of stomatitis (inflammatory edema beneath the covering mucosal epithelium) in the buccal mucosa was macroscopically observed and evaluated on a 4-point scale from 0 to 3 according to the edema score (Fig 3B). Subsequently, the buccal mucosal tissues, including the stomatitis area, were collected, divided into two parts, and used for genetic analysis and histological evaluation.

### Histological analysis

Collected tissues were fixed with 4% paraformaldehyde, and paraffin tissue blocks were prepared; sections of 4.5 µm thickness were cut using a microtome. The sections were stained with hematoxylin and eosin for routine histological evaluation, examined under a light microscope, and histomorphometric analyses were performed. Vessel density in the region directly beneath the ulcer was quantified per unit area at 100 × magnification. For inflammatory cell assessment, after imaging at 400 × magnification, three regions directly beneath the ulcer were randomly selected, and the numbers of neutrophils, lymphocytes, and plasma cells per unit area were quantified. The mean of these counts was considered the representative value for each animal. All results were summarized in the supplementary data (S2 Data).

### Reverse transcription-quantitative PCR (RT-qPCR)

Total RNA was extracted and purified from hamster tissues using TRI Reagent^®^ (Molecular Research Center, Inc., Cincinnati, OH, USA) after manual grinding of the tissues using a sterile grinding stick. The Applied Biosystems^®^ StepOne™ Real-time PCR System (Thermo Fisher Scientific, MA, USA) was used with One Step TB Green PrimeScript^TM^ RT-PCR Kit II (Perfect Real Time) (Takara Bio Inc. Shiga, Japan) following the manufacturer’s protocol. The primers used for this system were as follows: hamster *Tnf-α*, 5’-CTCCTTCCTGCTTGTGGGAG-3’ (sense) and 5’-GAGCCGATGATAGGGTTGGG-3’ (antisense); hamster *cyclooxygenase-2* (*Cox-2*), 5’- ATGACTGCCCAACTCCCTTG-3’ (sense) and 5’- ACACCTCTCCACCAATGACC-3’ (antisense); hamster *Il-6*, 5’- TCTTCTTGGGACTGCTGCTG-3’ (sense) and 5’- TGTTCGTCACAAACTCCAGGT-3’ (antisense); hamster *Il-1β*, 5’- TAGCTTTCCACAGCGATGAGA-3’ (sense) and 5’- GCTCTTGTTGAGGTCCTGCT-3’ (antisense); and hamster *glyceraldehyde-3-phosphate dehydrogenase* (*Gapdh*) as an internal standard, 5’-ACAGTCAAGGCTGAGAACGG-3’ (sense) and 5’-CAGGCGACATGTGAGATCCA-3’ (antisense).

### Cell culture

RT7 cells were cultured with GibcoTM Keratinocyte-SFM (Thermo Fisher Scientific, MA, USA) containing 25 µg/mL of bovine pituitary extract, 0.05 ng/mL of epidermal growth factor, 100 U/mL of penicillin, and 100 µg/mL of streptomycin. Cells were cultured under 5% CO₂ at 37 °C.

### PGE_2_ protein analysis by Enzyme-Linked Immuno Sorbent Assay (ELISA)

Confluently cultured RT7 cells in 96-well plates were washed with PBS, and 200 µL of Keratinocyte-SFM medium containing 10 µg/mL of *E. coli*-LPS and 0–0.00975 ppm of GL was added. After 6 h, the cell supernatant was collected and the expression of PGE_2_ protein was measured by competitive ELISA using the PGE_2_ ELISA Kit (#ADI-900–001, Enzo Life Sciences, Inc., NY, USA) according to the manufacturer’s protocol.

### Statistical analysis

Data are reported as mean ± standard deviation. For the evaluation of the time-course in the stomatitis-initiation model, one-way ANOVA followed by Dunnett’s multiple comparisons test was performed. Asterisks indicate significant differences compared with the Control (0 h) group (*p < 0.05). For all other experiments, one-way ANOVA followed by Tukey’s multiple comparison test was conducted. Means labeled with different letters were considered significant differences (*p* < 0.05). All statistical analyses were performed using GraphPad Prism (Version 10.6.0; GraphPad Software, San Diego, CA, USA). All statistical analysis results, including statistical significance, effect sizes, and confidence intervals, are summarized in the supplementary data (S1 Data).

## Results

### Healing-promoting effect of GL in a stomatitis-healing model

#### Macroscopic observation.

We used the previously established stomatitis-healing model [27] to verify the healing effect of GL on stomatitis. We injected 30 µL of 10% acetic acid into the right cheek pouch of the hamster, and on the following day, we observed a well-demarcated round white lesion, including an ulcer (defined as “wound”) (Fig 1). The wound size decreased over time. The area of the wound was compared with that in the stomatitis (ST)/physiological saline (PS) control group, which healed spontaneously.

**Fig 4:**
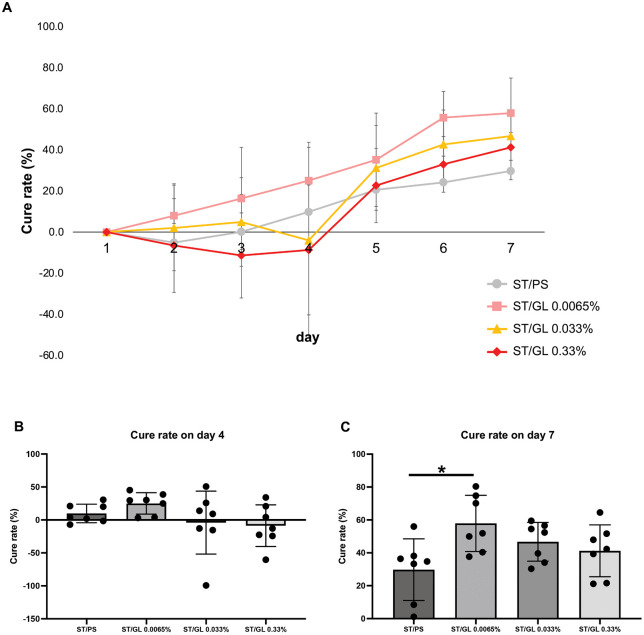
Changes in cure rate over time in the stomatitis-healing model. **(A)** The healing efficacy of each animal was evaluated by comparing the wound area over time. The cure rate was calculated using the following formula: Cure rate (%) = {(wound area on day 1 – wound area on day n) / wound area on day 1} × 100. Here, day n represents the wound area on day n after acetic acid injection. **(B)** Cure rate on day 4. **(C)** Cure rate on day 7. Data are presented as the mean ± standard deviation (n = 7 per group). Intergroup differences were analyzed using one-way ANOVA followed by Tukey’s multiple comparison test. Means labeled with different letters were considered significantly different (*p* < 0.05). Healthy: healthy negative control group. ST/PS: ST/PS-positive control group. ST/GL: ST/GL 0.0065-0.33% groups. ST: Stomatitis; PS: Physiological saline; GL: Glycyrrhizin.

The cure rate (%) was higher in the ST/GL 0.0065% group from day 2 than in the ST/PS group, and the cure rate increased significantly by day 7 (*p* = 0.0156). Meanwhile, compared with the ST/PS group, no significant increase in cure rates was observed in the ST/GL 0.033% and ST/GL 0.33% groups on day 7 (ST/GL 0.033%, *p* = 0.2239; ST/GL 0.33%, *p* = 0.5486) (Fig 4).

#### Histological findings.

**Fig 5:**
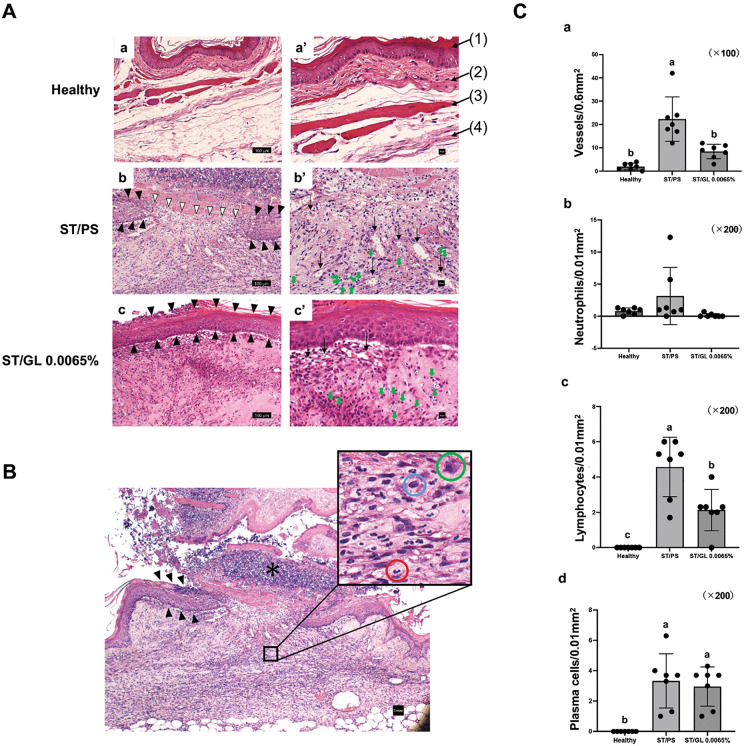
Histological findings of the Stomatitis-healing model. Histological findings on day 7 after stomatitis induction of the stomatitis-healing model (hematoxylin and eosin staining). **(A)** Healthy negative control group (a, a’), ST/PS-positive control group (b, b’), and ST/GL 0.0065% group (c, c’). a–c are under low magnification (scale bars: 100 µm); a’–c’ are under high magnification (scale bars: 10 µm). Healthy: The cheek pouch is composed of (1) flat and thin, regular keratinized stratified squamous epithelium, (2) dense submucosal connective tissue, (3) a muscular layer, and (4) loose connective tissue. No inflammatory cell infiltration was observed. ST/PS: The epithelial mucosal surface remains ulcerated (▽); granulation tissue with vessels (↓) and chronic inflammatory cell infiltration (green arrows) proliferating in the deeper portion of the ulcer base. ST/GL 0.0065%: New mucosal epithelium (▲) covering the ulcer surface. ST: Stomatitis; PS: Physiological saline; GL: Glycyrrhizin. **(B)** Histological findings of the ST/PS group. The thick exudate (*) covering the wound area, and the newly formed epithelium (▼) is elongated under thick exudate in the ulcer margin area. This figure is under low magnification (scale bars: 1 mm). Quantitative assessment was performed by counting the inflammatory cells present within the black frame (100 µm × 100 µm). Colored circles highlight neutrophils with segmented nuclei (red), lymphocytes characterized by large round nuclei and scant cytoplasm (blue), and plasma cells with eccentric nuclei and abundant basophilic cytoplasm, which are larger than lymphocytes (green). **(C)** Histomorphometric analysis was performed. The number of vessels (dilated vessels) within the region directly beneath the ulcer was quantified per unit area under 100 × magnification by visual inspection (a). Three regions directly beneath the ulcer were randomly selected, and the number of inflammatory cells (neutrophils; b, lymphocytes; c, and plasma cells; d) per unit area was quantified. The mean value of these counts was regarded as the representative value for each animal. Data are presented as the mean ± standard deviation (n = 7 per group). Intergroup differences were analyzed using one-way ANOVA followed by Tukey’s multiple comparison test. Means labeled with different letters were considered significantly different (*p* < 0.05).

We evaluated the healing-promoting effect of GL on wounds by comparing the histological findings of the wounds in the ST/PS and ST/GL 0.0065% groups on day 7. Healthy cheek pouches, as reported in a previous study [27], are composed of stratified squamous epithelium, dense connective tissue, muscle tissue, and loose connective tissue; no infiltration of inflammatory cells was observed in these layers (Figs 5Aa, 5Aa’).

The pathological findings of the wound in the ST/PS group revealed that the mucosal epithelial surface was ulcerated (▽) and covered with thick exudate (Fig 5B), and at the ulcer margin area, the newly formed mucosal epithelium (▲) was beginning to elongate between the granulation tissue and exudate. Additionally, in the ST/PS group, significant increases in granulation tissue with vessels (↓) and chronic inflammatory cell infiltration (lymphocytes and plasma cells) were observed in the deeper areas of the ulcer base compared with the Healthy group (*p* < 0.0001, *p* < 0.0001, *p* < 0.000327), indicating a transition to the healing phase (Figs 5Ab, 5Ab’, 5C).

By contrast, in the ST/GL 0.0065% group, which had the smallest wound size, a newly formed epithelium (▲) covered the wound surface (epithelialization). In the ST/GL 0.0065% group, compared with the ST/PS group, significant reductions in granulation tissue with chronic inflammatory cell infiltration (lymphocytes) and vessels were observed (*p* = 0.00313, *p* = 0.0009), whereas collagen fibers had increased (Figs 5Ac, 5Ac’, 5C). These results histologically confirmed the progression of wound healing in the GL groups.

**Fig 6:**
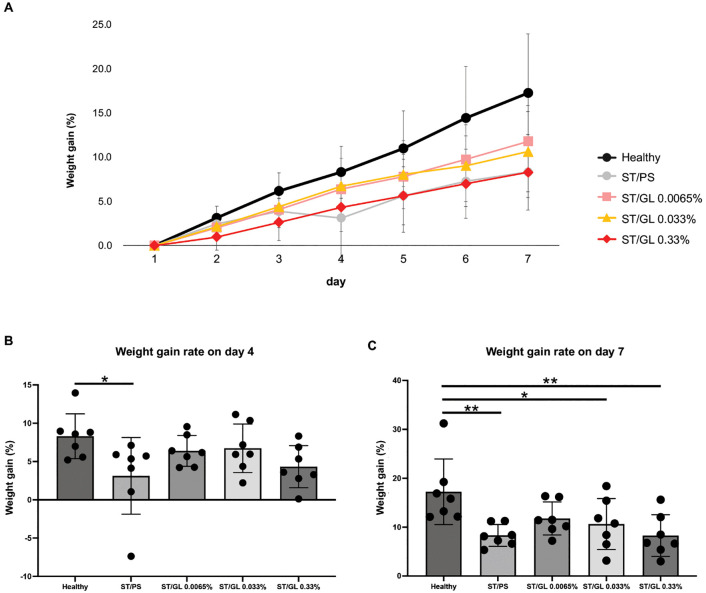
Weight gain rate (%) in the stomatitis-healing model. **(A)** The percentage of weight gain in each group was calculated based on the body weight on day 1. The following formula was used to determine the amount of weight gain from the day after acetic acid injection: Percent weight gain (%) = [(dn - d1)/d1] × 100, where d1 is the weight on 1 day after acetic acid injection, and dn is the weight recorded n days after acetic acid injection. **(B, C)** Weight gain rate on day 4 and day 7. Data are presented as the mean ± standard deviation (n = 7 per group) Intergroup differences were analyzed using one-way ANOVA followed by Tukey’s multiple comparison test. Means labeled with different letters were considered significantly different (*p* < 0.05). Healthy: healthy negative control group. ST/PS: ST/PS-positive control group. ST/GL: ST/GL 0.0065-0.33% groups. ST: Stomatitis; PS: Physiological saline; GL: Glycyrrhizin.

Fig 6 shows the indirect evaluation of stomatitis severity in the experimental groups based on the rate of weight gain. We previously reported that severe stomatitis causes severe pain, which leads to a decrease in food intake, ultimately resulting in weight loss, using the same stomatitis hamster model [27].

Compared with the healthy group, the ST/PS group showed a significant reduction in weight gain (*p* = 0.04926) (Figs 6A, 6B) at 4 days after stomatitis induction, and this was continuously suppressed until 7 days after (*p* = 0.00893) (Figs 6A, 6C). Moreover, at 7 days after induction, the ST/GL 0.33% group showed significant inhibition of weight gain, as did the ST/PS group (*p* = 0.00862) (Fig 6C). Conversely, the ST/GL 0.0065% and ST/GL 0.033% groups did not significantly suppress the rate of weight gain compared with the healthy group (Figs 6B, 6C). These results suggest that low concentrations of GL may promote stomatitis healing.

### Establishment of a novel stomatitis-initiation model

**Fig 7:**
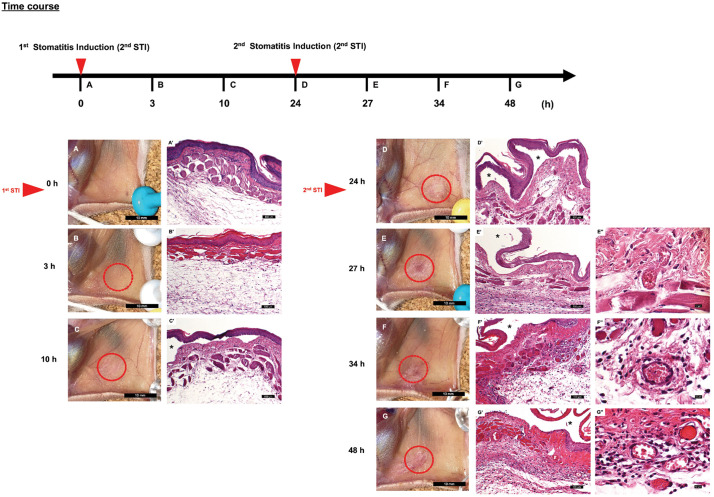
Time course of the macroscopic and histological findings in the stomatitis-initiation model. Healthy control (0 h, without stomatitis induction (STI)) **(A, A’)** and time points after stomatitis induction: 3 h after the first STI **(B, B’)**, 10 h after the first STI **(C, C’)**, 24 h after the first STI (just before the second STI) **(D, D’)**, 27 h (3 h after the second STI) **(E, E’)**, 34 h (10 h after the second STI) **(F, F’)**, 48 h (24 h after the second STI) **(G, G’)**. The white and red areas in the macroscopic findings correspond to regions in the histology where the epithelium is detached and cracked (*) and regions where vasodilation has occurred **(E**” **- G”)**, respectively. Hematoxylin and eosin staining. Scale bars are 10 mm **(A-G)**, 100 µm **(A’ – G’)**, and 10 µm **(E” - G’)**.

To evaluate the preventive effect of GL on stomatitis development, we established a stomatitis-initiation model, in which weak stomatitis (inflammatory edema) was induced by attaching filter paper impregnated with acetic acid to the buccal mucosa of hamsters. Macroscopic and histological changes in the stomatitis-initiation model were traced (Fig 7). In terms of macroscopic observations, no significant difference was noted between 0 and 3 h after the first stomatitis induction (STI) by acetic acid stimulation (Figs 7A, 7B). At 10 and 24 h after the first STI, an edematous lesion with a slightly rough and whitish mucosal surface was observed (Figs 7C, 7D). Because erythema, as initial characteristic of RAS was not observed, a second STI was performed 24 h after the first. The second STI led to the formation of a more prominent edematous lesion with erythema, and the degree of edema worsened over time (Figs 7E-7G).

**Fig 8:**
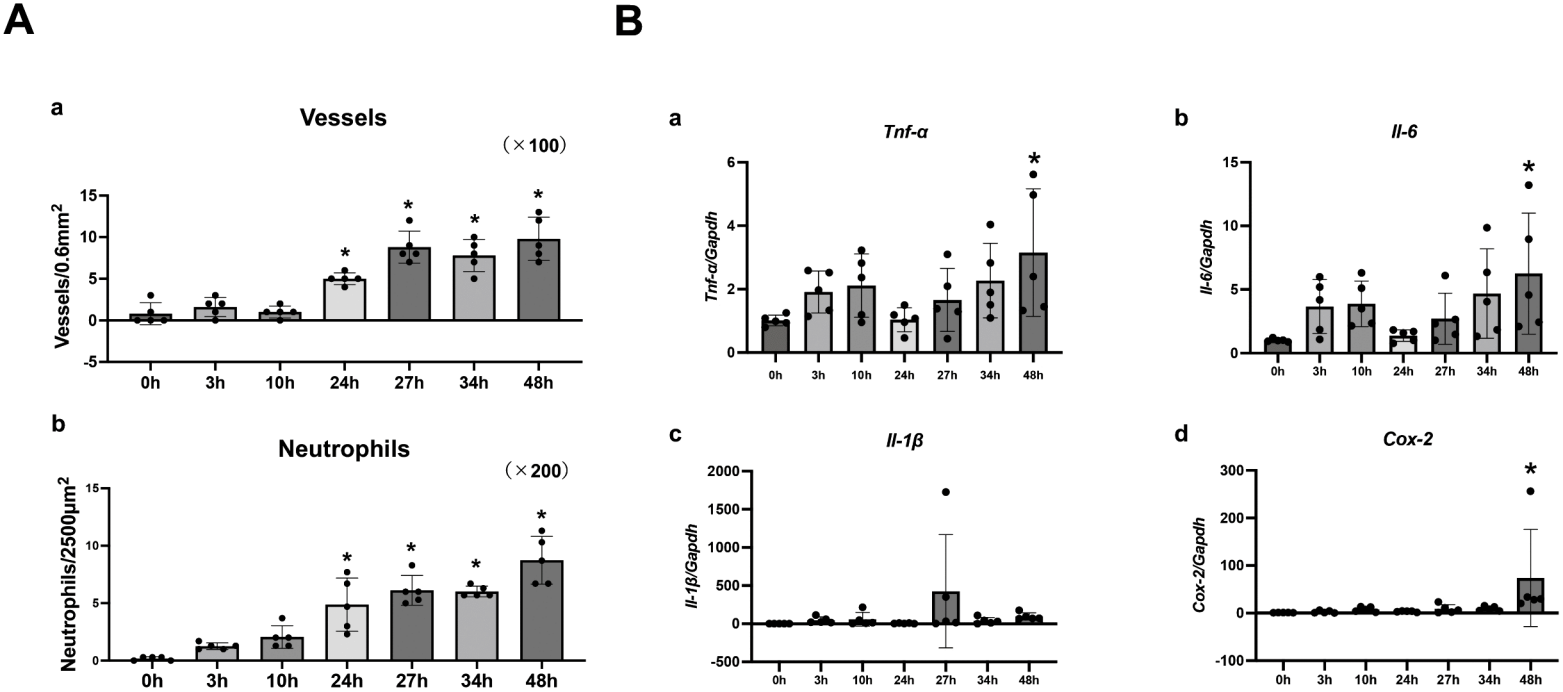
Time course of the histomorphometric analysis and gene expression analysis in the stomatitis-initiation model. **(A)** Histomorphometric analysis was performed. The number of vessels (dilated vessels) in the mucosal lesion induced by stomatitis was quantified per unit area under 100 × magnification by visual inspection (a). Three regions in the mucosal lesion induced by stomatitis were randomly selected, and the number of neutrophils per unit area was quantified (b). The mean value of these counts was regarded as the representative value for each animal. Data are presented as the mean ± standard deviation (n = 5 per group). **(B)** Time course of pro-inflammatory cytokine gene expression in the stomatitis-initiation model. Relative mRNA expression levels of *Tnf-α* (A)*, Il-6* (B)*, Il-1β* (C), and *Cox-2* (D) were analyzed using RT-qPCR. *Gapdh* was used as an internal control. Data are presented as means ± standard deviation (n = 5 per group). Intergroup differences were analyzed using one-way ANOVA followed by Dunnett’s multiple comparison test. Asterisks indicate significant differences compared with the Control (0 h) group (*p < 0.05).

Histologically, no significant difference was noted at 0 and 3 h after the first STI (Fig 4B’). At 10 and 24 h after the first STI, the epithelium was degenerated and detached from the connective tissue (Figs 7C’, 7D’). Compared with 0 h, the connective tissue surface showed a significant increase in the number of vessels and neutrophils at time points after 24 h (*p* < 0.000001, *p* < 0.000001) (Figs 8Aa, 8Ab). At 27 h (3 h after the second STI), vasodilation was observed in the subepithelial connective tissue (Figs 7E’, 7E”). At 34 and 48 h (10 and 24 h after the second STI, respectively), epithelial degeneration and subepithelial damage were prominent. The nuclei of the desquamated epithelium were indistinct, suggesting necrosis. In addition, a thick layer of fibrinopurulent exudate was observed on the connective tissue surface, and progressive neutrophil infiltration along with numerous dilated vessels extended into the muscular layer (Figs 7F’, 7F”, 7G’, 7G”).

The results of the time-course analysis of the changes in the expression of inflammation-related genes associated with RAS in the novel stomatitis-initiation model showed that the expression of *cyclooxygenase-2* (*Cox-2*)*, tumor necrosis factor-α* (*Tnf-α*) and *interleukin-6* (*IL-6*) mRNA were significantly increased at 48 h after STI (24 h after the second STI) (*p* = 0.0295, 0.0185 and 0.0167, respectively) (Fig 8B).

### Effects of GL in the stomatitis-initiation model

#### Macroscopic observation.

**Fig 9:**
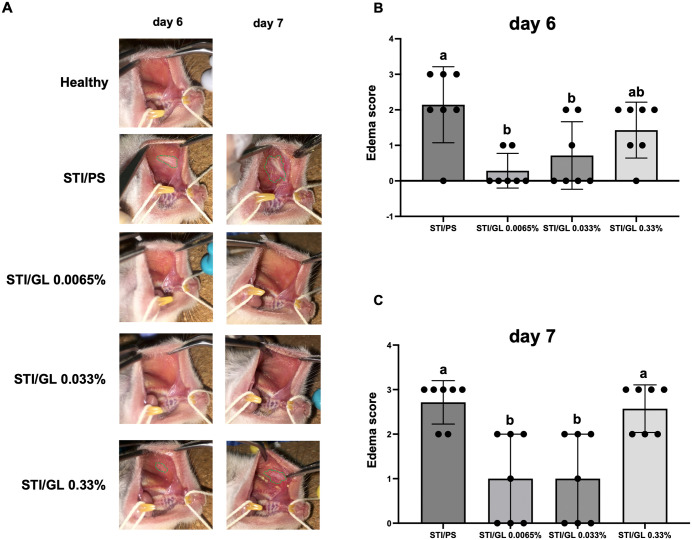
Edema score in the stomatitis-initiation model. The degree of edema was visually observed and scored on a 4-point scale from 0 to 3 according to the edema score (Fig 3B). Data are presented as mean ± standard deviation (n = 7 per group). **(A)** Macroscopic findings on day 6 and day 7. Edema is indicated by the green dotted line. **(B)** Edema score on day 6. **(C)** Edema score on day 7. Intergroup differences were analyzed using one-way ANOVA followed by Tukey’s multiple comparison test. Means labeled with different letters were considered significantly different (p < 0.05). STI/PS: STI/PS-positive control group. STI/GL: STI/GL 0.0065-0.33% groups. STI: Stomatitis induction; PS: Physiological saline; GL: Glycyrrhizin.

GL solution was continuously applied to the buccal mucosa of the hamsters daily during the experimental period. Edematous lesions caused by acetic acid stimulation were observed macroscopically on day 6 (Fig 9A) and day 7 (Fig 9B). The degree of edema was scored on a 4-point scale from 0 to 3 (Fig 3B). On day 7 (1 day after the second STI), the degree of inflammatory edema was significantly suppressed in the STI/0.0065% GL and STI/0.033% GL groups compared with that in the STI/PS control group (*p* = 0.002526, *p* = 0.002526). Conversely, the STI/0.33% GL group showed no significant difference in the edema score compared with the STI/PS control group, indicating that low concentrations (0.0065–0.033%) of GL have a preventive effect on stomatitis but not high concentrations (0.33%) (Fig 9B). These findings reveal that the effective concentration range of the preventive effect of GL on stomatitis may differ from that of its healing-promotion effect.

#### Histological findings.

**Fig 10:**
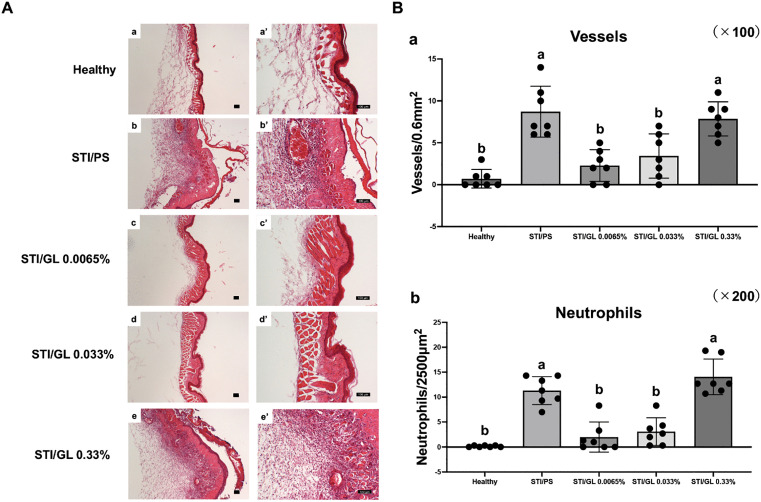
Histological findings of the stomatitis-initiation model. Histological findings on day 7 (1 day after the second STI). **(A)** Healthy negative control group (a). STI/PS-positive control group (b). ST/GL 0.0065-0.33% groups (c-e). a–e are under low magnification (scale bars: 0.1 mm); a’–e’ are under high magnification (scale bars: 100 µm). STI: Stomatitis induction; PS: Physiological saline; GL: Glycyrrhizin. **(B)** Histomorphometric analysis was performed. The number of vessels (dilated vessels) in the mucosal lesion induced by stomatitis was quantified per unit area under 100 × magnification by visual inspection (a). Three regions in the mucosal lesion induced by stomatitis were randomly selected, and the number of neutrophils per unit area was quantified (b). The mean value of these counts was regarded as the representative value for each animal. Data are presented as the mean ± standard deviation (n = 6 per group). Intergroup differences were analyzed using one-way ANOVA followed by Tukey’s multiple comparison test. Means labeled with different letters were considered significantly different (*p* < 0.05).

In this model, the pathology of the edematous lesion was confirmed on day 7 (1 day after the second STI). Histopathologically, desquamation of the degenerated epithelium and deposition of fibrinous exudate in the subepithelial connective tissue and muscular layer were observed in the STI/PS group. In the deeper portion, severe capillary dilatation with hyperemia, edema, and neutrophil infiltration were evident (Figs 10Aa, 10Aa’, 10Ab, 10Ab’), and the numbers of vessels and neutrophils were significantly increased compared with the Healthy group (*p* = 0.000002, *p* < 0.000001) (Figs 10Ba, 10Bb). By contrast, these findings were not noticeable in the two lower concentration groups (STI/GL 0.0065% group and STI/GL 0.033% group), and the development of inflammatory edema was suppressed (Figs 10Ac, 10Ac’, 10Ad, 10Ad’). Surprisingly, the high-concentration group (STI/GL 0.33% group) showed histopathological findings comparable to the STI/PS group, including vasodilatation, inflammatory cell infiltration, and edema (Figs 10Ae, 10Ae’).

### Gene expression analysis

**Fig 11:**
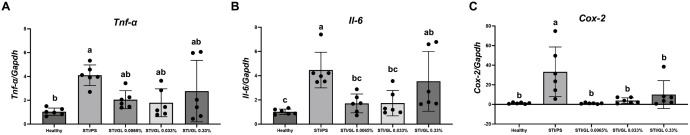
Gene expression analysis using RT-qPCR. Total RNA was extracted and purified from the buccal mucosa on day 7, and RT-qPCR was performed. Data are presented as mean ± standard deviation (n = 6 per group). Intergroup differences were analyzed using one-way ANOVA followed by Tukey’s multiple comparison test. Means labeled with different letters were considered significantly different (*p* < 0.05). Healthy: only physiological saline (PS) was applied to healthy buccal mucosa; STI (stomatitis induction)/PS: PS applied daily to buccal mucosa with STI, STI/GL 0.33%, STI/GL 0.033%, and STI/GL 0.0065%: glycyrrhizin (GL) solutions applied daily to buccal mucosa with STI.

RT-qPCR was performed to identify changes in the expression levels of *Tnf*-α, *Il-6,* and *Cox-2* mRNA at the site of inflammatory edema. Compared with that of the STI/PS group, the expression of *Tnf*-α mRNA was suppressed in the STI/GL 0.033% and STI/GL 0.0065% groups, but the difference was not significant (Fig 11A). Compared with that of the STI/PS group, the expression of *Il-6* mRNA was significantly suppressed in the STI/GL 0.033% and STI/GL 0.0065% groups (*p* = 0.0201 and 0.0189), but compared with that of the Healthy group, it remained significantly upregulated in the STI/GL 0.33% group (*p* = 0.0366) (Fig 11B). Furthermore, compared with that of the STI/PS group, the expression of *Cox-2* mRNA was significantly suppressed in all three STI/GL groups (STI/GL 0.0065%; *p* = 0.0022, STI/GL 0.033%; *p* = 0.0055, and STI/GL 0.33%; *p* = 0.0369) (Fig 11C).

### PGE_2_ expression analysis in RT7 cells by ELISA

**Fig 12:**
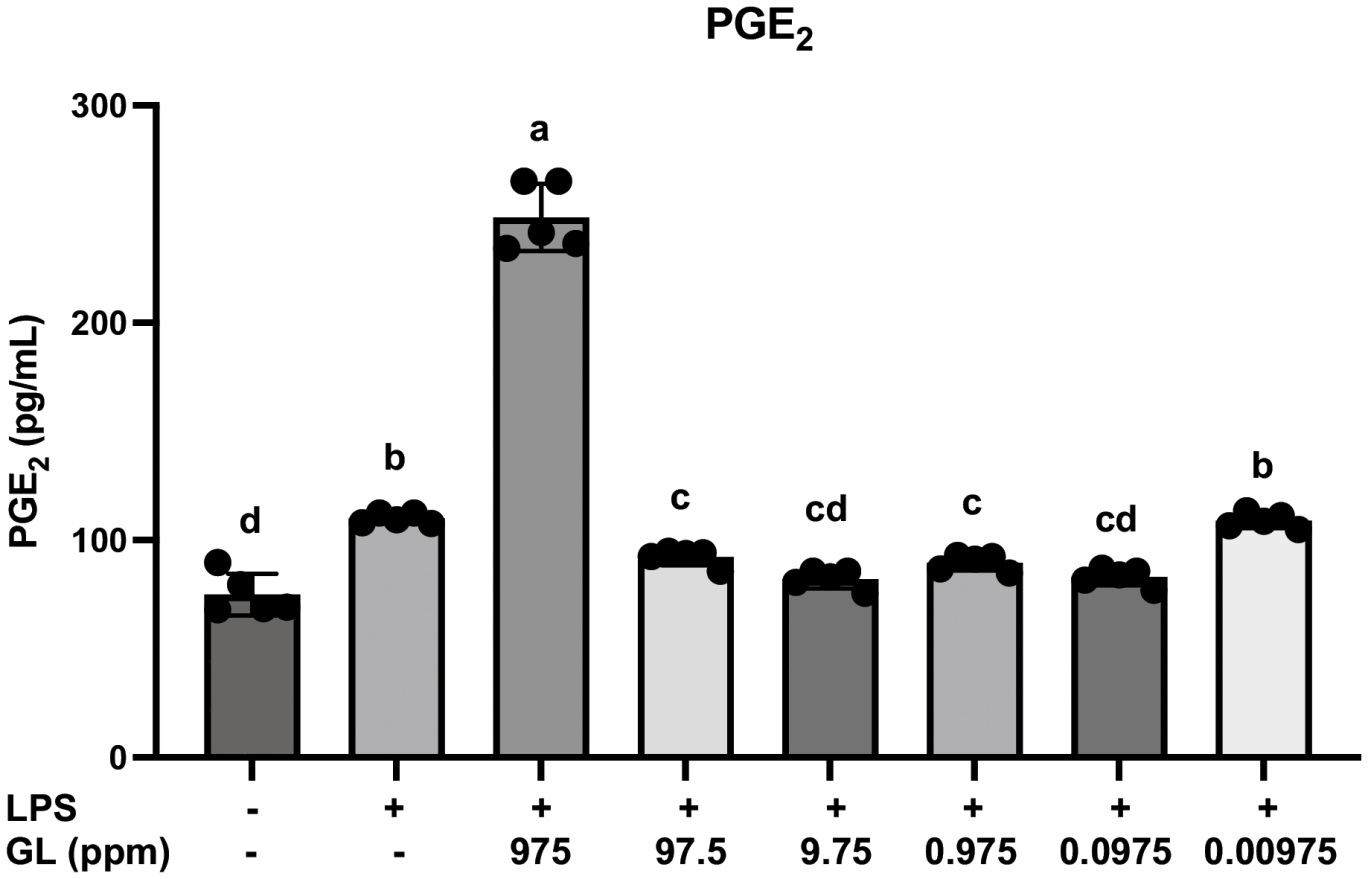
PGE_2_ protein expression analysis in RT7 cells by ELISA. PGE_2_ protein levels in RT7 cells were measured by ELISA. Data are presented as mean ± standard error of the mean (n = 6 per group). GL: glycyrrhizin. Intergroup differences were analyzed using one-way ANOVA followed by Tukey’s multiple comparison test. Means labeled with different letters were considered significantly different (*p* < 0.05).

Previous studies reported that the incidence of bacterial infections in oral aphthae is high [30] and that *E. coli* colonization may cause RAS [31]. Mucosal bacterial infection affects innate immunity and inflammation through PGE_2_ production. We hypothesized the existence of an optimal concentration of GL capable of inhibiting PGE_2_ production by the mucosal epithelium. To verify this, the effect of various GL concentrations on PGE_2_ production was examined in human oral keratinocytes (RT7) challenged with *E. coli*-LPS. GL concentrations in the range of 97.5–0.0975 ppm suppressed the production of PGE_2_ elevated by LPS stimulation, whereas at high GL concentrations (975 ppm), PGE_2_ production was significantly upregulated (Fig 12).

## Discussion

By implementing the stomatitis-healing model and the newly established stomatitis-initiation model, we report that GL may effectively promote the healing and prevention of oral stomatitis.

Healing-promoting effects in stomatitis have been reported for plant-derived anti-inflammatory agents—such as curcumin, Aloe vera gel, and propolis—as well as for synthetic/semi-synthetic medications; however, evidence for prophylactic efficacy (i.e., preventing ulcer onset/recurrence) remains limited and heterogeneous [32–38].

GL is a triterpene isolated from licorice roots and rhizomes [15] and is widely used in pharmaceuticals and cosmetics, due to its anti-inflammatory and antioxidant properties [39–41]. Based on prior studies indicating that GL could potentially prevent enterocolitis in rats via the reduction of NF-κB p65 and p38 mitogen-activated protein kinase expression [42], we hypothesized that GL might exhibit both preventive and healing effects on stomatitis. In this study, we demonstrated the effect of GL on stomatitis using two animal models.

The developmental process of stomatitis is classified into five phases: initiation (I), primary damage response (II), signal amplification (III), ulceration (IV), and healing (V) [28]. Direct intradermal injection of acetic acid rapidly leads to severe ulcer formation (phase IV) covered by thick exudate; therefore, we used this model to assess the effect of GL on stomatitis healing.

In the stomatitis-initiation model, the filter paper with acetic acid attached to the mucosal surface stimulated the epithelial cells (I), causing degeneration and necrosis (II), leading to the production of cytokines and inflammatory mediators in the epithelium and subepithelial area. This resulted in moderate vasodilatation, inflammatory edema and inflammatory cell infiltration (III), and finally, epithelial separation but not exposure of connective tissue (transition to ulceration phase). Thus, this model is suitable for assessing the preventive effect of GL on oral stomatitis formation.

Following the protocol in our previous study [27], we replicated human oral stomatitis-like lesions covered by thick exudate, associated with notable inflammatory cell infiltration at the ulcer base. To evaluate the healing-promotion effect, we measured the clearly demarcated circular white lesions that included ulcers (termed “wounds”) formed by intramucosal acetic acid injection into the cheek pouch. The daily application of GL to the wound area immediately after wound formation demonstrated GL’s ability to promote wound healing and indicated a tendency to restore the reduced hamster weight gain rate. On day 7, histological evaluation revealed an inflammatory cell infiltration, predominantly composed of neutrophils from the ulcer surface to the muscle layer of the PS group. Conversely, the GL group, especially the 0.0065% GL group, showed the smallest wound size, which was completely covered with a newly formed mucosal epithelium, and revealed a reduction in dilated vessels, inflammatory cells and an increase in collagen formation. These results provide histological confirmation of the accelerating effect of GL on wound healing.

The stomatitis-healing model, which rapidly progresses to phase IV (ulcerations), is dedicated to the evaluation of the healing-promotion effect and is unsuitable for evaluating the stomatitis prevention effect of GL. Therefore, a new stomatitis-initiation model that corresponds to phases I–III, was constructed to evaluate the stomatitis prevention effect. Macroscopically, we confirmed that the acetic acid-immersed paper-applied area exhibits edematous lesions with a partially reddish surface. Furthermore, the model induces a significant increase in *Tnf-*α, *Il-6,* and *Cox-2* mRNA expression at the gene level, and vasodilation with hyperemia, inflammatory cell infiltration, and inflammatory edema in the subepithelial area at the macroscopic level.

Through sustained daily prophylactic administration of GL before the induction of stomatitis, a significant reduction in edema score was observed in the low-concentration GL groups (0.0065% and 0.033%). By contrast, no preventive effects were observed in the high-concentration GL group (0.33%). Microscopic observations revealed the separation of the necrotic covering epithelium, a superficial fibrinous exudate layer, and severe inflammatory cell infiltration, similar to those in the PS group.

Moreover, genetic analysis revealed a significant elevation in *Il-6* mRNA expression in edematous lesions. Notably, sustained application of lower GL concentrations effectively inhibited the increase in *Il-6* expression. Conversely, higher GL concentrations did not significantly reduce *Il-6* mRNA expression. IL-6 is produced by a variety of cells, including macrophages, B cells, T cells, and fibroblasts, and plays important roles in regulating hematopoiesis, immune cell activation, and inflammation [43]. Additionally, high levels of IL-6 have been detected in the circulation of patients with the active phase of RAS [44]. Taking these findings together, GL at low concentrations may effectively prevent stomatitis by inhibiting the IL-6-mediated inflammatory pathway.

In this study, we also confirmed that GL significantly suppresses the expression of *Cox-2*. Previous studies reported that the incidence of bacterial infection is high in oral aphthae [30]. In addition, it has been suggested that the cause of RAS may be the colonization of *E. coli* [31]. PGE_2_ can modulate inflammatory reactions via the EP2/4 receptor through its regulation of TNF-α and IL-6 production [45]. Hence, the presence of high GL concentrations in the oral mucosa in inflammatory conditions may result in the exacerbation of inflammation via the excessive production of PGE_2_.

The present study showed that high concentrations of GL may enhance the expression of PGE_2_ by oral keratinocytes stimulated with LPS. Although GL is broadly reported to suppress HMGB1–TLR4–NF-κB signaling [46], dose- and context-dependency should be considered. At higher concentrations, GL may trigger cellular stress responses (e.g., reactive oxygen species) [47], and/or skew macrophage polarization toward a pro-inflammatory M1 phenotype, which can secondarily activate the NF-κB/COX-2 axis [48]. These mechanisms provide a plausible explanation for the paradoxical increase in PGE₂ and the lack of *Il-6* suppression observed in our high-dose group.

This study has some limitations. One limitation is the inability to investigate in detail the mechanisms by which high concentrations of GL may adversely affect the prevention of stomatitis. The precise mechanisms underlying the action of GL can be elucidated through gene expression analyses in the future. Another limitation is that this study is based on cellular and animal experiments. Further clinical trials are required and that findings may not fully translate to human pathology due to interspecies differences. We expect that forthcoming clinical trials will verify the preventive effect of GL on RAS.

In this study, we set and evaluated three GL concentrations within the range previously established in stomatitis therapeutics and oral-care products. Therefore, the present findings may serve as preclinical evidence supporting the effective concentration range of GL for future clinical applications.

## Conclusion

This study established a novel stomatitis-initiation model that successfully reproduces the early morphological and inflammatory stages preceding ulcer formation. This innovative model not only enhances our understanding of the initial events in stomatitis pathogenesis but also provides a valuable tool for developing effective preventive and therapeutic strategies for RAS.

The findings obtained using the stomatitis-healing and initiation models demonstrated for the first time that GL may not only have a healing-promotion effect but also a significant preventive effect on stomatitis. In addition, limiting the concentration is important for achieving optimal effects of GL, which has the potential to improve the quality of life of patients with RAS.

## Supporting information

S1 Table Body weight of hamsters used in the experiment. Body weight of each hamster at baseline in all experimental groups. The individual data shown in the gray-shaded area were previously published (Shiba et al., 2024; https://doi.org/10.1371/journal.pone.0313747), and do not impact the present findings.
(XLSX)

S1 Data Summary of statistical analysis results. All statistical analysis results are provided in this file, including statistical significance, effect sizes, and confidence intervals for all comparisons conducted in the study.
(XLSX)

S2 Data Histomorphometric analysis of ulcerated tissues. This dataset summarizes the histological and histomorphometric analyses performed on the ulcerated buccal tissues. Vessel density in the region directly beneath the ulcer was quantified per unit area at 100 × magnification. For inflammatory cell analysis, after imaging at 400 × magnification, three random regions beneath the ulcer were evaluated, and the numbers of neutrophils, lymphocytes, and plasma cells per unit area were recorded. The mean value for each animal was used as the representative value.
(XLSX)
